# Transient Expression of GATA3 in Hematopoietic Stem Cells Facilitates Helper Innate Lymphoid Cell Differentiation

**DOI:** 10.3389/fimmu.2019.00510

**Published:** 2019-03-21

**Authors:** Dejene M. Tufa, Ashley M. Yingst, Tyler Shank, Seonhui Shim, George Devon Trahan, Jessica Lake, Renee Woods, Kenneth L. Jones, Michael R. Verneris

**Affiliations:** ^1^Department of Pediatric Hematology, Oncology and BMT, School of Medicine, University of Colorado, Aurora, CO, United States; ^2^Department of Children's Cancer and Blood Disorders, Children's Hospital of Colorado, Aurora, CO, United States

**Keywords:** GATA3, HSCs, helper ILCs, differentiation, electroporation

## Abstract

Helper Innate lymphoid cells (ILCs) are tissue resident lymphocytes that play a critical role in a number of biological processes. Several transcription factors are required for the differentiation of hematopoietic stem cells (HSCs) into ILCs. Recent studies demonstrate GATA3 as a transcriptional regulator that plays an essential role in ILC development. We aimed to modulate the differentiation of human cord blood-derived CD34^+^ cells into ILCs by transient and ectopic expression of mRNA encoding transcription factors known to be important for ILC lineage differentiation, including GATA3, TOX, NFIL3, ID2, and RORγt. Using this experimental protocol, only GATA3 significantly modulated HSCs to differentiate into helper ILCs. Transient overexpression of GATA3 drove the emergence of CD34^+^α4β7^+^ early ILC progenitors during the first few days of culture. These ILC progenitors further acquired IL-7Rα and CD117 to give rise to immediate ILC precursors. In support of these findings, analysis of the genes induced by GATA3 in HSCs showed an upregulation of those associated with ILC development. Moreover, we show GATA3 also acts on more committed progenitors and significantly shifts the differentiation of progenitors away from the ILC1/NK lineage to the ILC2 and ILC3 lineage. In summary, transient overexpression of GATA3 mRNA in CD34^+^ HSCs enhances the differentiation of HSCs into the helper ILC lineages, at the expense of NK cell development.

## Introduction

Innate lymphoid cells (ILCs) are group of tissue-resident lymphocytes that have been grouped into three different cell types according to their transcription factor expression and cytokine production following stimulation (1, 2). Group 1 ILCs, which include natural killer (NK) cells, express T-bet and produce IFN-γ and some are cytotoxic upon activation (3, 4). Group 2 ILCs express GATA3 and produce IL-5 and IL-13 upon stimulation, whereas group 3 ILCs express RORγt and produce IL-22 following stimulation (5–9). Recent data suggests that ILCS are the innate counterparts of T lymphocytes and functionally, helper ILCs have similarity to various CD4^+^ helper T cell lineages, whereas NK cells resemble CD8^+^ cytotoxic T cells (10).

According to murine studies, all ILCs develop from common lymphoid progenitors (CLP), which further differentiates into the common ILC progenitor (CILP). The CILP in turn, differentiates into an NK progenitor (NKP), as well as the common “helper” innate lymphoid cell precursor (CHILP). Eventually, the NK progenitors differentiate to NK cells, whereas the CHILP differentiates to all ILCs such as ILC1, ILC2, and ILC3, but not NK cells (1, 2, 11, 12).

In both murine models and human correlative studies, helper ILCs have been found to have beneficial functions in a variety of disease settings requiring tissue repair and homeostasis. For instance, through the production of IL-22, host ILC3s protect the murine thymus from radiation-induced injury that occurs during hematopoietic cell transplantation (HCT) conditioning (13). Similarly, ILC3-derived IL-22 acts on intestinal stem cells, thereby protecting the gastrointestinal tract from graft vs. host disease (GVHD) (14, 15). Through different mechanisms, adoptive transfer of murine ILC2 cells protects against the induction and severity of GVHD in mouse models (16). Indeed, in studies of HCT recipients, higher numbers of activated ILCs, both before and after transplantation were correlated with less GVHD and mucositis (17). Also of interest is that ILC3s concentrate in tumor tissue of patients with low stage lung cancer where they are associated with the generation of tertiary lymphoid structures, perhaps facilitating antitumor immune responses (18). Given these attributes, adoptive transfer of ILCs might be therapeutically beneficial in certain clinical settings, however given their paucity in the circulation and tissue resident location, alternative approaches would be necessary to produce such cells for therapeutic indications.

The generation of ILCs from HSCs requires several transcription factors that act at distinct and often overlapping stages of differentiation (19). Several recent reports using murine models demonstrate that multiple transcription factors such as GATA3, ID2, RORγt, NFIL3, and TOX are required for ILC differentiation (19–24). Transduction of Lin^−^CD127^+^CD294^−^ cells by viruses expressing GATA3 resulted in the development of functionally active ILC2 cells (25). Recently, murine studies show that loss of GATA3 leads to a deficiency in not only ILC2's, but instead, all ILC subsets were diminished, suggesting that GATA3 may be important at an earlier stage of ILC development and serve as a master regulator of ILC development (26–28). Here we designed an approach to *in vitro* generate ILCs by ectopically expressing different transcription factors (GATA3, ID2, RORγt, NFIL3, and TOX) in UCB-derived HSCs. We report that transient overexpression of GATA3 mRNA in human HSCs favors their differentiation into CILPs and then to give rise to all helper ILCs, at the expense of NK cells.

## Materials and Methods

### Isolation and Expansion of CD34^+^ HSCs

Cord blood mononuclear cells were isolated from UCB by density gradient centrifugation using Lymphoprep (Stemcell). The CD34^+^ HSCs were positively enriched from UCB-derived PBMCs using MACS CD34^+^ enrichment kit (Miltenyi). The cells (purity, >95%) were suspended (5 × 10^4^ cells/ml) in Stemspan serum free expansion medium cell culture media (Stemcell) supplemented with 1% penicillin + streptomycin, SCF (100 ng/ml, R&D), Flt3L (100 ng/ml, Stemcell), TPO (50 ng/ml, R&D) and LDL (10 ug/ml, Stemcell) and cultured in 24 well plate for 5 days of expansion. After 5 days of expansion the cells were expanded ~3-fold while the proportion of CD34^+^ cells remained >95% (Supplementary Figure 1).

### Preparation of mRNAs

Six genes (GATA3, ID2, RORC, NFIL3, TOX, and GFP) were considered for this study and the DNA for the reference sequences of each gene were obtained from Integrated DNA Technologies (Coralville, IA) as gblocks. Each DNA sequence corresponding to a particular gene was designed to contain the T7 promoter in the 5′ end, 5′UTR, 3′UTR, and primer sites for Gibson's cloning. The fragments were cloned into the pCoofy40 vector (Addgene plasmid # 44006, a gift from Sabine Suppmann) using Gibson cloning. Briefly, the digested vector and the gblocks were combined in equimolar ratios and incubated at 50°C using a thermocycler. Following the assembly, the vector containing the genes of interest were transformed into top 10 competent cells (New England Biolabs). The plasmid was then purified from a colony of *E. coli* using EZNA plasmid extraction kit (Omega biotech). The isolated plasmid was digested with restriction enzymes to confirm inserts of the correct size. To prove the sequence, the plasmid was sequenced using a classic Sanger sequencing protocol.

To produce *in vitro* transcribed mRNA, the fragment containing the overall portion of the gblock, excluding the portion of the vector, was amplified by PCR from a plasmid DNA using a forward primer: ttggaccctcgtacagaagctaatacg and reverse: 120t-cttcctactcaggctttattcaaagacca (a primer that contains long poly A tail of repeating T sequences for 120 bases). The PCR product was cleaned using a Qiagen PCR reaction cleaning kit according to the manufacturer's protocol. The capped mRNA was produced from 0.5 ug clean DNA using the T7-mMESSAGE mMACHINE *in vitro* transcription kit (Thermofisher Scientific). The mRNA was cleaned using Qiagen RNA cleanup Kit. The concentration of mRNA was analyzed and its integrity and size were also checked using Experion RNA StdSens Analysis kit (Bio-Rad).

### Transfection and Differentiation of CD34^+^ HSCs

After 5 days of expansion, CD34^+^ HSCs were considered for further differentiation experiments. Additionally, FACS sorted α4β7^−^CD34^+^ cells were isolated from expanded CD34^+^ HSCs (Supplementary Figure 2). At Day 5 of expansion, CD34^+^ HSCs were transfected by mRNAs corresponding to various transcription factors using nucleofector kits for human CD34^+^ cells (Lonza) according to the company procedure. Briefly, 1 × 10^6^ cells were centrifuged to remove the media, resuspended in 100 ul transfection buffer and 3 ug GATA3, ID2, RORC, NFIL3, TOX or control GFP mRNA was added. Cell suspension containing the mRNA was then added to cuvette followed by electroporation using amaxa 4D nucleofector apparatus. Following electroporation, cells were suspended in a previously described B0 differentiation media (29) supplemented with SCF (20 ng/ml, R&D), IL-3 (5 ng/ml, Stemcell), IL-7 (20 ng/ml, R&D), IL-15 (10 ng/ml, NIH), IL-23 (10 ng/ml, R&D) and Flt3L (10 ng/ml, Stemcell). Cells were then cultured in the presence or absence of pre-plated and irradiated EL08.1D2 stromal cells. After a week of culture, IL-3 was excluded from the B0 differentiation media supplements. Culturing, maintaining and preparation of irradiated stromal layer of EL08.1D2 cells or OP9 on 96 well plate culture was as described earlier (30). For plating cells on the stromal cells, 100 cells were plated per well of 96 well plates on the irradiated EL08.1D2 cells in 150 ul B0 differentiation media. Alternatively, cells were also plated off stroma using 96 well u-bottom plate and 1 × 10^3^ cells were cultured per well. For single cell cloning experiments, either GATA3 or mock mRNA transfected CD34^+^ cells were FACS sorted as single cells per well of 96 well plate on the irradiated EL08.1D2 or OP9 cells followed by 28 days of differentiation.

### mRNA-seq Analysis

RNA was extracted from 5 days expanded and GATA3 or control mRNA transfected UCB-derived CD34^+^ HSCs. Total of 1 × 10^6^ cells, 5 days after electroporation, were used for RNA extraction using Rneasy mini kit (Qiagen) according to manufacturer's protocol and the purity and concentration of RNA was measured on a Nano drop (Thermofisher Scientific). Five hundred nanogram of RNA was used to prepare Illumina HiSeq libraries according to manufacturer's instructions for the TruSeq Stranded RNA kit (Illumina Inc.). The mRNA template libraries were then sequenced as single pass 50 bp reads on the Illumina HiSeq 4000 platform at the University of Colorado's Genomics Core Facility. The derived sequences were analyzed by applying a custom computational pipeline gSNAP, Cufflinks and R for sequence alignment and ascertainment of differential gene expression (31). Briefly, reads generated were aligned to the human reference genome (hg19) by gSNAP (32), expression (FPKM) was derived by Cufflinks (33), and R was used to analyze differential expression using a paired *T*-test in GATA vs. Control. Genes significant at a FDR <0.05 were submitted to pathway analysis using Ingenuity Pathway Analysis (Qiagen) to identify genes of interest that were modified downstream of GATA3. Additionally, as heterogeneity within donors was seen, treatment effects were analyzed independently by donor to determine differences in their response. Heatmaps, principal component analysis (PCA) plot and UpSet plots were generated to illustrate the results.

### Quantitative PCR (qPCR)

Transfection efficiency was analyzed by using qPCR 2 days after electroporation. The expressions of GATA3, ID2, RORC, NFIL3, and TOX in GATA3 transfected cells were compared to mock transfected cells using qPCR. To perform qPCR, total RNAs were extracted from cells using Rneasy kit (Qiagen) and reverse transcribed into cDNAs using advanced iSuperscript cDNA synthesis kit (Bio-Rad). The qPCR reaction was performed in duplicates using Taqman gene expression assays (Thermofisher Scientific) and run by StepOnePlus real-time PCR system (Thermofisher Scientific) using the standard qPCR protocols. Analysis was done using applied biosystems StepOne analysis software and the quantitative expression of the target genes were determined after normalizing to the expression of GAPDH.

### Western Blot and Flow Cytometry Analysis

Western blotting or flow cytometry was used to analyze the level of GATA3, ID2, RORγt, NFIL3, and TOX protein expression 48 h post mRNA transfection of the cells. Differentiation of α4β7^+^, CD117^+^, or CD127^+^ ILC progenitors and mature ILCs were also analyzed using flow cytometry. Moreover, viability of cells was analyzed using flow cytometry with the help of fixable viability dye eFluor 780 (eBioscience). Expression of surface receptor was determined using the following monoclonal antibodies: anti-CD3-PerCp5.5 (clone HIT3a), anti-CD11c-PerCp5.5 (clone Bu15), anti-D14-PerCp5.5 (clone 63D3), anti-CD19-PerCp5.5 (clone HIB19), anti-CD123-PerCp5.5 (clone 6H6), anti-CD127-PE (clone A019D5), anti-CD294-BV421/BV605 (clone BM16), anti-CD336-APC (clone P44-8), and anti-mouse IgG-FITC/PE (clone Poly4053) (all from Biolegend); anti-CD11a-FITC (clone G43-25B), anti-CD34-PE/APC (clone 4H11), anti-CD45-APC/PE (clone HI30), anti-CD56-BV421/BV605 (clone NCAM16.2), anti-CD94-PerCp5.5/FITC (clone HP-3D9), anti-CD117-PE-Cy7 (clone 104D2), and anti-CD161-PE (clone DX12) (all from BD Biosciences); and anti-α4β7 (Cat#11718, from NIH). As negative controls fluorochrome conjugated isotype-matched antibodies from the respective companies were utilized. Flow cytometry acquisitions were performed using LSR II and data were analyzed using Flowjo (BD Biosciences) or Kaluza (Beckman Coulter) analysis software.

## Results

### Ectopic Overexpression of GATA3 mRNA in CD34^+^ HSCs Modulates Gene Expression

**Figure 1:**
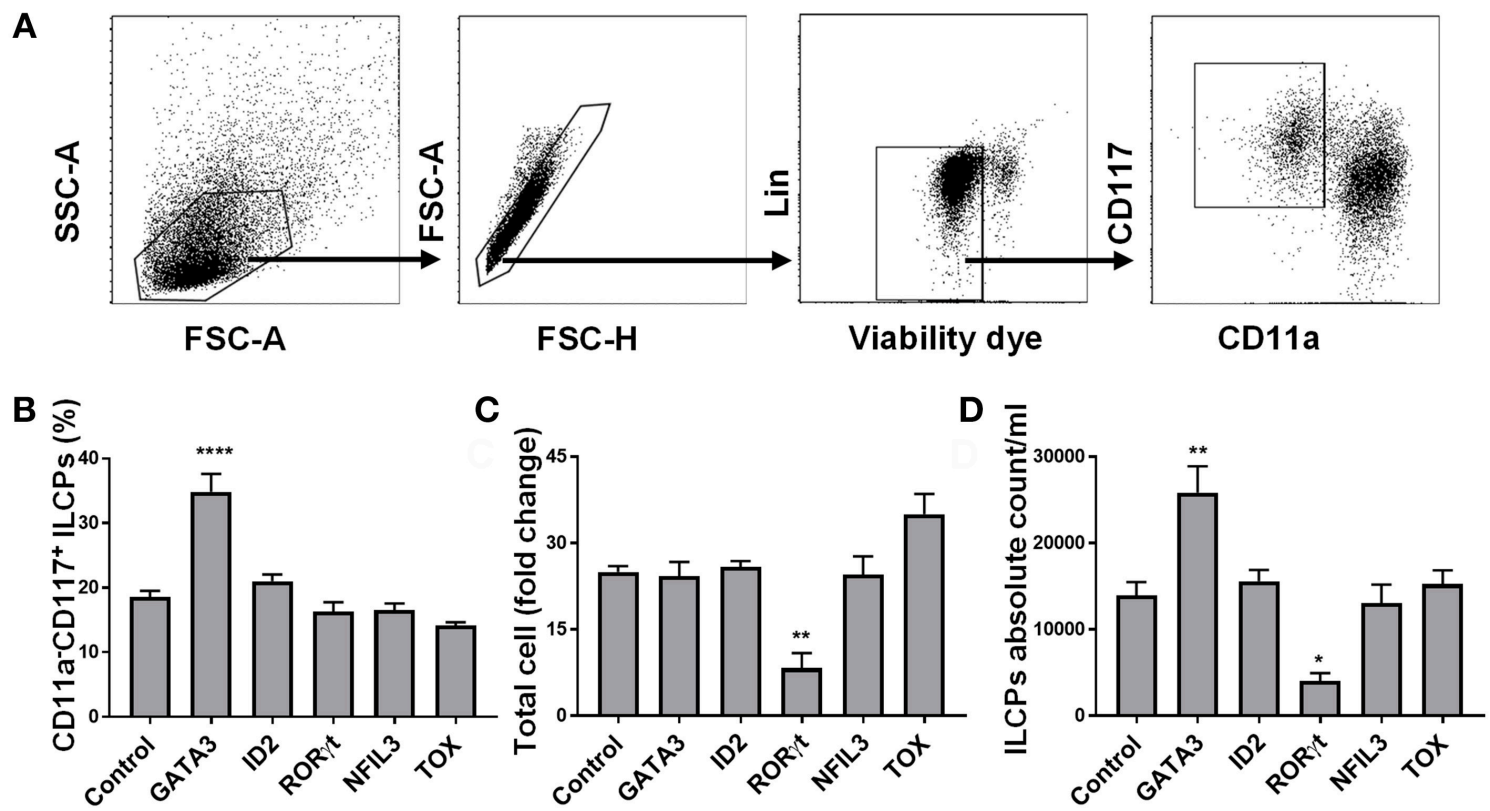
GATA3 but not ID2, RORγt, NFIL3, or TOX influences the differentiation of ILCs from HSCs. Five-day expanded UCB CD34^+^ HSCs were transfected with either GATA3, ID2, RORC, NFIL3, TOX or scrambled control mRNAs and differentiated under conditions that favor ILCPs development. **(A)** Gating strategy of ILCPs, 14-days differentiating HSCs. **(B)** Differentiating HSCs were stained for the surface CD11a and CD117 at day 14 post electroporation and the percentage of CD117^+^CD11a^−^ ILCPs is shown in bar graph (*n* = 4/group). **(C)** Total counts of differentiating HSCs were performed at day 14 of culture and fold change is depicted (*n* = 4/group). **(D)** Absolute counts of CD117^+^CD11a^−^ ILCPs in 1 mL of culture media is depicted (*n* = 4/group). **(B–D)** Data are shown as mean ± SD, One-way ANOVA and significance comparing to the control is shown (*p < 0.01; **p < 0.001; ****p < 0.0001).

We aimed to determine whether transient expression of transcription factors previously shown to play a key role in ILC development might skew HSC differentiation into the ILC lineage under the influence of instructive cytokines. To test this hypothesis, we cloned the genes for GATA3, ID2, NFIL3, RORC, TOX, and GFP (control) and *in vitro* transcribed these into mRNA which was electroporated into CD34^+^ cells that had been cultured for 5 days (to obtain sufficient cell numbers for transfection). We used flow cytometry and tested the expression of GATA3, ID2, NFIL3, RORγt, and TOX 48 h after electroporating *in vitro* transcribed GATA3, ID2, NFIL3, RORC, and TOX mRNAs, respectively, into CD34^+^ cells and found higher protein expression in the electroporated cells compared to controls (Supplementary Figure 3). When the electroporated cells were cultured for a total of 14 additional days, the only transcription factor that impacted the percentage and absolute number of ILC precursors (defined as Lin^−^CD11a^−^CD117^+^) (34– 36) was GATA3 (Figures 1A–D). Therefore, we focused our efforts and aimed to further understand how GATA3 influences ILC development from CD34^+^ HSCs.

**Figure 2:**
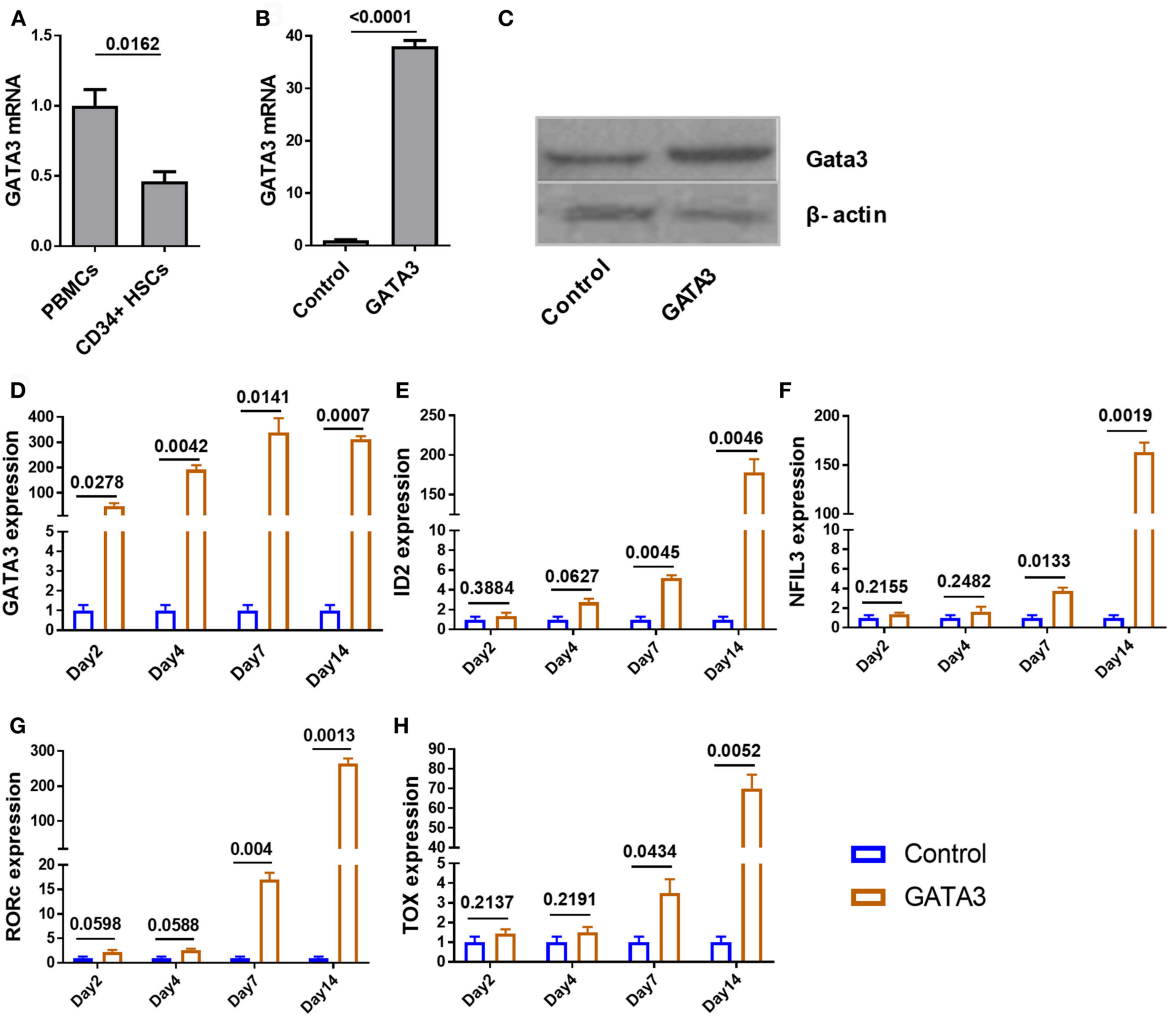
Ectopic expression of GATA3 in CD34^+^ HSCs has no immediate influence on other ILC related transcription factors. Five-day expanded UCB CD34^+^ HSCs were transfected with either GATA3 mRNA (GATA3) or scrambled mRNA (Control) and RNAs were extracted at various time points to perform qPCR. To assess GATA3 protein, cells were also lysed 48 h post transfection. **(A)** The quantitative expression of GATA3 mRNA in UCB CD34^+^ HSCs is shown relative to its expression in UCB mononuclear cells after normalizing to the expression of GAPDH (*n* = 4/group). **(B)** The quantitative expression of GATA3 mRNA in GATA3 transfected cells is shown relative to control cells after normalizing to the expression of GAPDH (*n* = 5/group). **(C)** Western blot of GATA3 protein from the cell lysates at 48 h following GATA3 or control mRNA transfection. **(D,H)** The quantitative expression of genes at each time point in GATA3 transfected cells is shown relative to its expression in control cells after normalizing to the expression of GAPDH (*n* = 3/group). **(A,B,D–H)** Data are shown as means ± SD, *p*-values are depicted and paired two-tailed *t*-tests.

To investigate the expression of GATA3 at steady state in CD34^+^ cells, we used qPCR and found that GATA3 mRNA expression in CD34^+^ HSCs is about two times lower when compared to bulk cord blood-mononuclear cells (Figure 2A). We next tested the relative GATA3 mRNA expression 48 h after electroporating *in vitro* transcribed GATA3 mRNA into CD34^+^ cells and found ~35-fold higher expression, which also translated into higher protein expression at 48 h (Figures 2B,C). Using qPCR we found that GATA3 transfection was associated with a significant increase in the expression of ILC-related transcription factors including ID2, NFIL3, RORC, and TOX starting at seven days after transfection, suggesting that GATA3 initiates a genetic program that leads to ILC development (Figures 2D–H).

**Figure 3:**
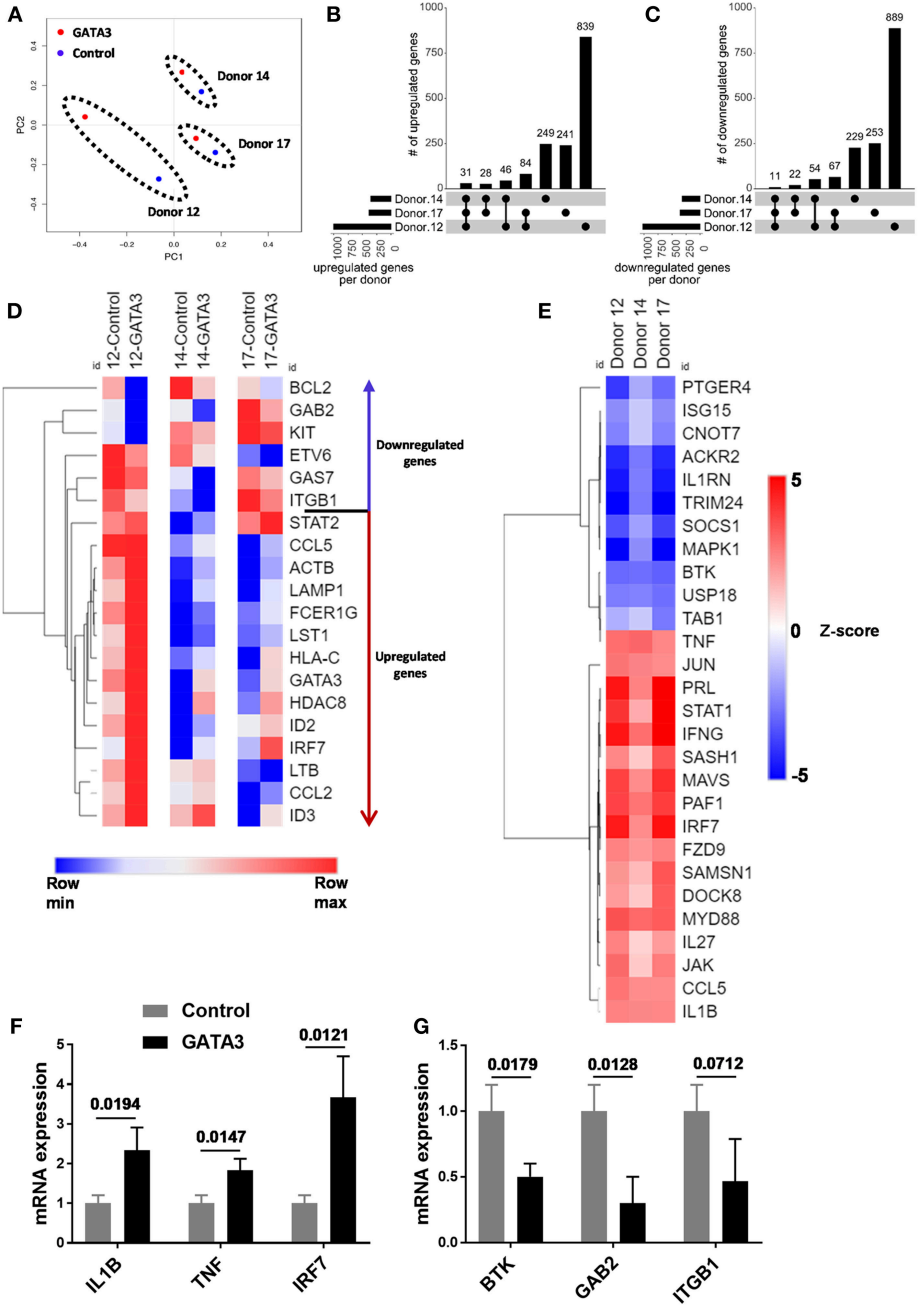
GATA3 influences the gene expression profile of CD34^+^ HSCs. Five-day expanded UCB CD34^+^ HSCs were transfected with either GATA3 mRNA (GATA3) or scrambled mRNA (control) and RNA was extracted after 5 days of culture and qPCR or mRNA sequencing was performed for 3 independent donors across GATA3 transfected and control transfected cells. **(A)** The principal component analysis of global gene expression was performed in R and the result is shown as PCA plot. **(B,C)** The number of upregulated **(B)** and downregulated **(C)** genes, based on mRNA expression within each donor, as well as the overlap between donors are displayed as an UpSet plot. **(D)** Heat map showing log transformed differential expression for each donor pair (GATA3 vs. control) for genes altered by GATA3. **(E)** Significantly regulated genes by GATA3 mRNA were considered for each donors to perform upstream analysis using IPA. A heat map showing activation z-scores for statistically significant upstream regulators. **(F,G)** The quantitative expression of genes in GATA3 transfected cells is shown relative to its expression in control cells after normalizing to the expression of GAPDH (*n* = 3/group), data are shown as mean ± SD, paired two-tailed *t*-tests and *p*-values are depicted.

To further investigate the impact of transient GATA3 expression we performed RNA sequencing to analyze transcript changes 5 days after HSC transfection. Despite major genetic differences in the three UCB CD34^+^ donors tested, a principal component analysis (PCA) showed similar effects of GATA3 mRNA transfection. As shown in Figure 3A, the transient overexpression of GATA3 mRNA led to coordinated changes in gene regulation, consistently shifting the global transcription of each donors in a similar manner. GATA3 significantly upregulated over 240 genes (28–84 in at least 2 donors and 31 in common for all donors), while it downregulated >220 genes (22–67 in at least 2 donors and 11 in common for all donors) (Figures 3B,C and Supplementary Figure 4). LTB, LST1, ID2, ID3, IRF7, ITGB1, BCL2, KIT, and GAB2 were some of the ILC-related genes modulated by GATA3 overexpression (Figure 3D). Moreover, upstream analysis using ingenuity pathway analysis (IPA) showed that GATA3 modulated the transcription of several innate lymphocyte related genes including activation of CCL5, IL1B, IL-27, IRF7, MAVS, and TNF, whereas GATA3 inhibited BTK, USP18, CNOT7, and SOCS1 (Figure 3E). To validate these observations, we performed qPCR and found upregulation of IL1B, TNF, IRF7 and a downregulation in BTK, GAB2, and ITGB1 in HSCs transfected with GATA-3 mRNA relative controls (Figures 3F,G).

### GATA3 Overexpression in CD34^+^ HSCs Induces the Development of ILC Progenitors

**Figure 4:**
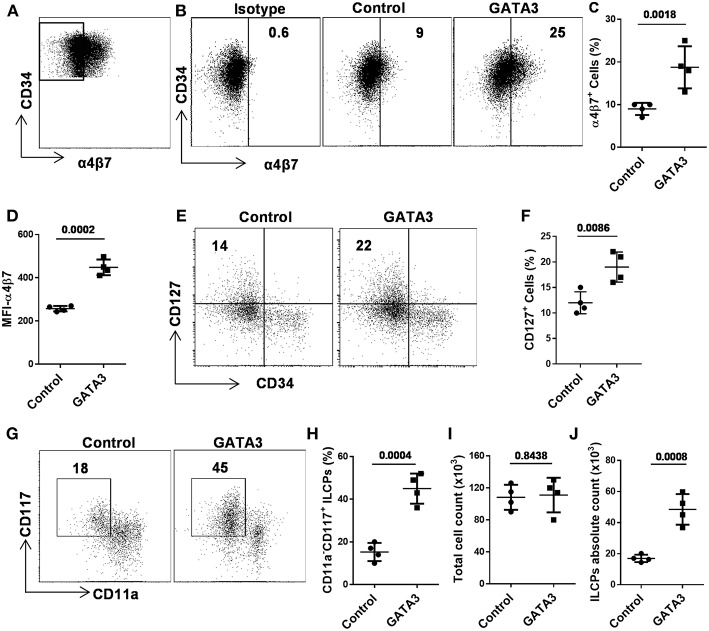
GATA3 induces the development of ILC progenitors. Five-day expanded UCB CD34^+^α4β7^−^ HSCs were transfected with either GATA3 mRNA (GATA3) or scrambled mRNA (control) and differentiated under conditions that favor the development of ILCs **(A)** Gating strategy, 5-days expanded CD34^+^α4β7^−^ HSCs were sorted by FACS. **(B)** CD34^+^α4β7^−^ HSCs were stained for the surface α4β7 at day 7 post electroporation and dot plot is shown (*n* = 4). Values represent the percentage of α4β7^+^ cells. **(C,D)** CD34^+^α4β7^−^ HSCs were stained for the surface α4β7 at day 7 post electroporation and the percentage **(C)** and MFI **(D)** of α4β7^+^ cells is shown (*n* = 4/group). **(E)** CD34^+^α4β7^−^ HSCs were stained for the surface CD127 at day 14 post electroporation and dot plot is shown (*n* = 4). Values represent the percentage of CD127^+^ cells. **(F)** CD34^+^α4β7^−^ HSCs were stained for the surface CD127 at day 14 post electroporation and the percentage of CD127^+^ cells is shown in bar graph (*n* = 4/group). **(G)** CD34^+^α4β7^−^ HSCs were stained for the surface CD11a and CD117 at day 14 post electroporation and dot plot is shown (*n* = 4). Values represent the percentage of CD117^+^CD11a^−^ ILCPs. **(H)** CD34^+^α4β7^−^ HSCs were stained for the surface CD11a and CD117 at day 14 post electroporation and the percentage of CD117^+^CD11a^−^ ILCPs is shown in bar graph (*n* = 4/group). **(I)** Total counts of transfected and differentiating HSCs were performed at day 14 of culture and live cells in 1 mL of culture media is depicted (*n* = 4/group). **(J)** Absolute counts of CD117^+^CD11a^−^ ILCPs in 1 mL of culture media is depicted (*n* = 4/group). **(C,D,F,H–J)**, Data are shown as means ± SD, and *p*-values are depicted and paired two-tailed *t*-tests.

Recent murine studies report that integrin α4β7 marks early ILCPs with the capacity to develop into all groups of ILCs (12, 20, 24, 28). In order to study the effect of GATA3 on the development of α4β7 expressing progenitors, we sorted CD34^+^ cells that lacked this integrin receptor (i.e., CD34^+^α4β7^−^ cells) after 5 days of culture (Figure 4A and Supplementary Figure 2), and transfected them with GATA3 or control/scramble mRNA. GATA3 mRNA electroporation into sorted CD34^+^α4β7^−^ HSCs significantly induces the generation of CD34^+^α4β7^+^ progenitors a week after electroporation (Figures 4B–D). Previously, our data and that of others, demonstrated that the ILC precursors express both tyrosine-protein kinase Kit (CD117) and interleukin-7 receptor-α (CD127) (34–36). Accordingly, electroporation of GATA3 mRNA into sorted CD34^+^α4β7^−^ HSCs significantly induced the development of CD127 expressing cells 2 weeks after electroporation (Figures 4E,F). In addition, overexpression of GATA3 in CD34^+^α4β7^−^ HSCs resulted in the generation of CD11a^−^CD117^+^ ILC precursors, and the proportion of these cells were significantly higher in samples transfected with GATA3 mRNA, but not with control mRNA (Figures 4G,H). The total cell count after 2 weeks of culture remained similar between the GATA3 mRNA transfected cells and control mRNA transfected cells (Figure 4I). Therefore, the absolute number of the ILC precursors were significantly increased in the GATA3 mRNA transfected group compared to controls (Figure 4J).

### GATA3 Overexpression in CD34^+^ HSCs Augments the Development of ILC2s and ILC3s and Abrogates the Development of ILC1/NK Cells

**Figure 5:**
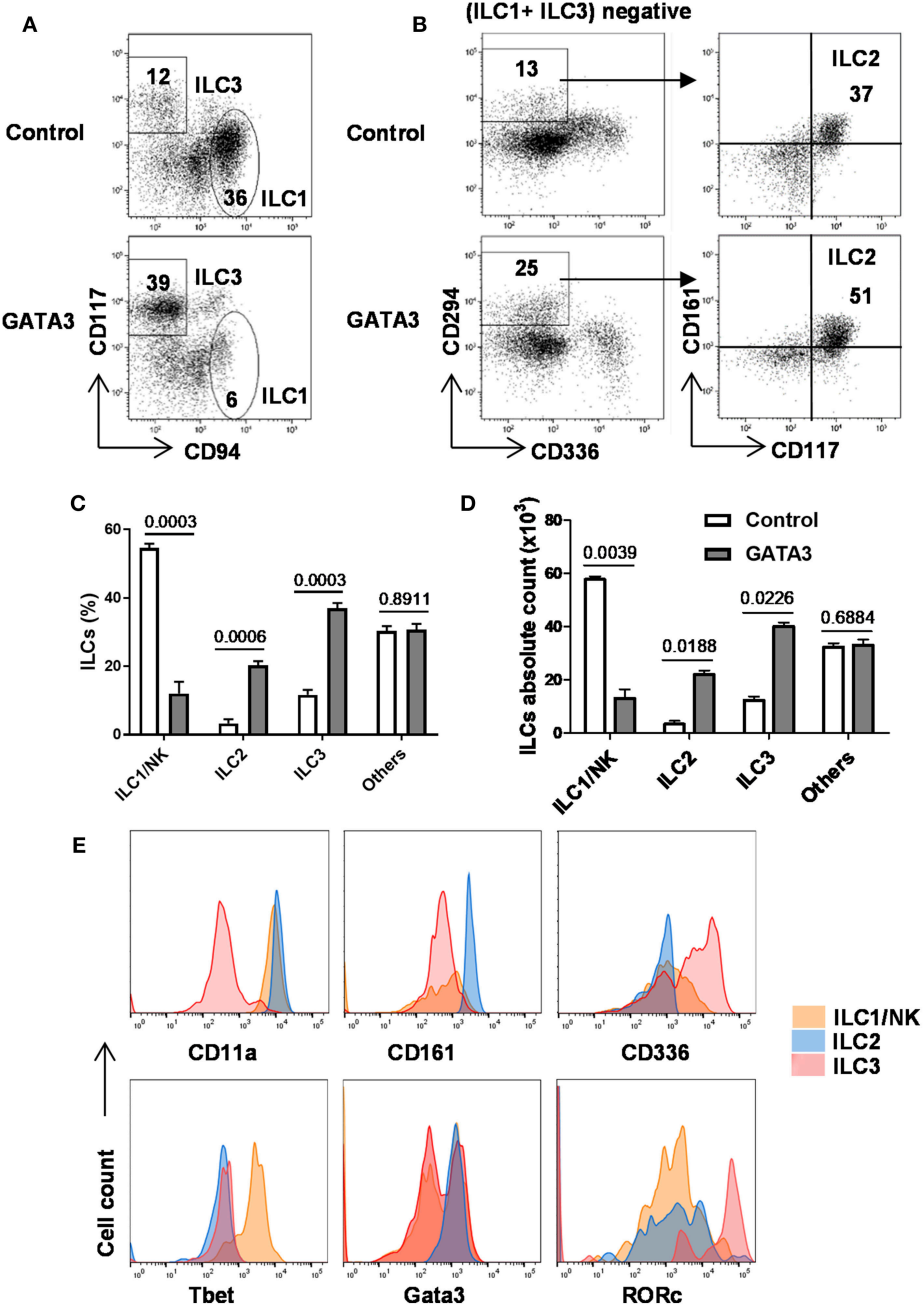
GATA3 induces the generation of ILC2 and ILC3 at the expenses of ILC1/NK cells. Five-day expanded cord-blood derived CD34^+^ HSCs were transfected with either GATA3 mRNA (GATA3) or control mRNA (Control) and then differentiated under conditions that favor the development of ILCs (see methods). **(A,B)** Both control (upper row) and GATA3 (lower row) mRNA transfected CD34^+^ HSCs were stained for ILCs surface markers at day 28 of differentiation culture and CD94^+^ ILC1/NK and CD117^+^ ILC3 **(A)**, and CD294^+^ ILC2 **(B)** from a representative donor are shown in dot plots (*n* = 6). Values represent the percentages of ILCs in the culture. **(C,D)** Differentiating CD34^+^ HSCs were stained for ILCs surface markers at day 28 of culture and the percentage **(C)** or absolute number **(D)** of CD94^+^ ILC1/NK, CD294^+^ ILC2, and CD117^+^ ILC3 are shown in bar graphs (*n* = 6/group). **(E)** Differentiating CD34^+^ HSCs were stained for surface CD11a, CD161, CD336, and intracellular Tbet, Gata3, RORγt at day 28 of culture and the representative histograms are shown (*n* = 6/group). **(C,D)** Data are shown as means ± SD, *p*-values are depicted and paired two-tailed t-tests.

As above, GATA3 induced the generation of CD34^+^α4β7^+^ progenitors, previously shown in mice to have the potential to give rise to all ILC lineages (12, 20, 24, 28). Therefore, we studied the generation of all ILCs in the culture after GATA3 mRNA electroporation. Different surface and intracellular markers have been used to distinguish ILCs into their lineages (1, 2, 37). Accordingly, cells that expressed Tbet, CD56, and CD94 were assigned as ILC1s, co-expression of Gata3, CD161, and CD294 defined ILC2 cells, and ILC3s were distinguished as cells expressing RORγt, CD56, CD117, and CD161 (1, 7, 34). The expressions of CD56 and CD336 in mature CD117^+^ ILC3s distinguish these cells from the CD117^+^ ILCPs (Supplementary Figure 5). Analysis of ILC content in fully differentiated cultures (day 28) using these surface markers show that compared to controls, transfection of GATA3 mRNA enhanced the differentiation of CD294^+^ ILC2 and CD117^+^ ILC3, while it antagonized the development of CD94^+^ ILC1 (NK cells) from CD34^+^ HSCs (Figures 5A–D). Similar to previous studies (1, 7, 34), our data also confirm expression of Tbet and CD11a by ILC1s, Gata3, and CD161 by ILC2s, and RORγt and CD336 by ILC3s (Figure 5E). Therefore, in addition to induction of CD34^+^α4β7^+^ progenitors from CD34^+^ HSCs, GATA3 enhances the generation of helper ILCs, but not that of NK cells from the progenitors.

### Single Cell Cloning Shows GATA3 Acts on Committed Precursors

**Figure 6:**
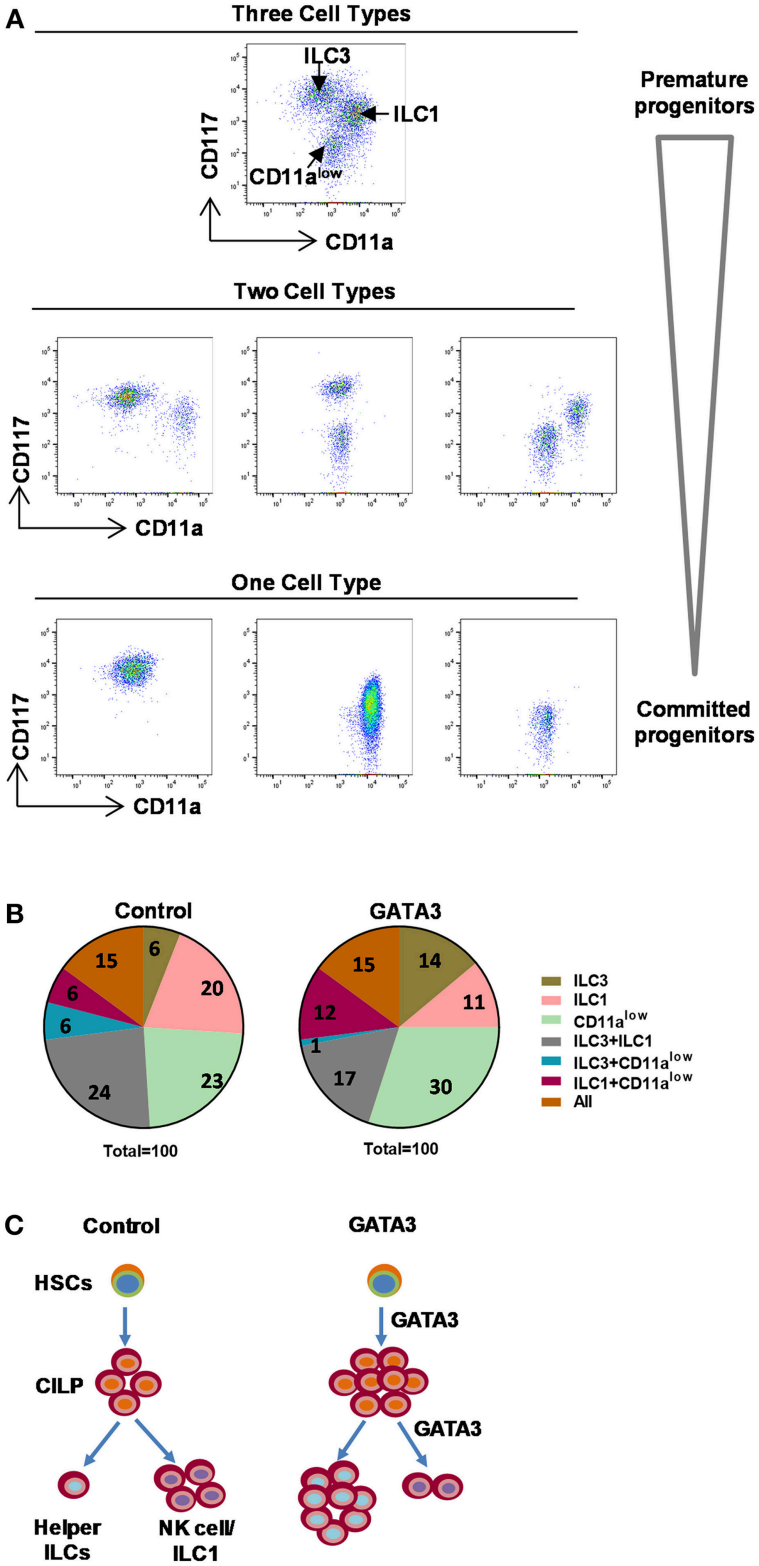
Single cell cloning demonstrated GATA3 acts on committed progenitors. Either mock or GATA3 mRNA transfected CD34^+^ HSCs were single cell FACS sorted and differentiated for 28 days on pre-plated and irradiated EL08.1D2 stromal cell line in 96 well plates (39/384 for mock transfected and 42/384 for GATA3 transfected wells supported cell growth). **(A)** Cells were stained for ILCs surface markers at day 28 of culture and the representative dot plots show that single cells differentiated to either CD11a^+^CD94^+^ ILC1, CD11a^−^CD117^+^ ILC3, CD11a^low^CD117^−^ cells, a combination of two cell subsets or a combination of three cell subsets. **(B)** Cells were stained for ILCs surface markers at day 28 of culture and the percentage of wells showing either CD11a^+^CD94^+^ ILC1, CD11a^−^CD117^+^ ILC3, CD11a^low^CD117^−^ cells or a combination of two or three cell subsets are shown in parts of whole graphs. **(C)** GATA3 induces the generation of ILCPs from HSCs, and the differentiation of helper ILC lineage from committed progenitors. A schematic presentation.

With the aim of understanding the stage of differentiation at which GATA3 acts to modulate ILC development, we designed a single cell cloning experiment where, expanded polyclonal CD34^+^ cells (containing HSCs of varying stages of development) were transfected with either GATA3 or control mRNA. Following transfection, single cells were FACS sorted onto pre-plated and irradiated EL08.1D2 or OP9 feeder cells with media containing cytokines (29, 30). We have previously observed that the EL08.1D2 feeder cells do not support CD294^+^ ILC2 growth (unpublished) and while this is a limitation, it is the most robust system to study ILC development and therefore affords single cell analysis experiments. After 4 weeks of differentiation, cell proliferation was detected in 11% (42/380) and 10.3% (39/380) of the wells seeded with a single GATA3 or control mRNA transfected cell, respectively. The 4 week differentiated cells were analyzed for ILC development and three subsets of cells were identified (CD11a^+^CD117^low^, CD11a^−^CD117^+^, and CD11a^low^CD117^−^) (Figure 6A). The CD11a^+^CD117^low^ cells comprise group 1 ILCs/NKs (Figure 5), the CD11a^−^CD117^+^ cells contain ILC3s (Figure 5), while CD11a^low^CD117^−^ cells expressed myeloid markers such as CD15 and CD16 (data not shown). In both the GATA3 mRNA transfected and control conditions 15% of the wells contained all the three of the above cells subsets (Figure 6B, orange). For the wells with GATA3 or control transfected cells, 30 and 36% of wells showed a combination of two of the three subsets (Figure 6B). Only one cell type was detected in 55% and 49% of the GATA3 and control wells, respectively (Figure 6B). However, GATA3 transfected conditions showed a doubling of the wells that specifically differentiated into ILC3s, whereas there was a nearly 50% reduction in the number of wells that grew into group 1 ILCs (Figure 6B, olive and pink). Single cell culture on OP9 demonstrated that GATA3 transfected conditions comparing to control showed increase in the number of wells that differentiated into ILC2s and ILC3s (Supplementary Figure 6). Thus, this single cell cloning data shows that GATA3 not only induces differentiation of HSCs to CILP, but also likely acts on more committed HSC progenitors favoring ILC3 differentiation at the cost of group 1 ILC development (Figure 6C).

## Discussion

Murine ILCs are derived from a common lymphoid progenitor that give rise to a common ILC progenitor (CILP), which in turn has the capacity to differentiate into all helper ILC subtypes (1, 2). In humans, the trajectory that an HSC takes during ILC development is only partially understood and the course of differentiation has been questioned as to whether or not it is similar to that of mice (38). Previously, it has been reported in mice that CILPs enrich within α4β7^+^CD117^+^ compartment of CD34^+^ HSCs (12, 20, 28) and cells with this same phenotype are present in human secondary lymphoid tissue and give rise to ILC3's *in vitro* (39). In this study, we evaluated whether mRNA transfection of transcription factors could skew the development of UCB-derived CD34^+^ cells into the ILC lineage. Using a panel of mRNAs for GATA3, ID2, RORγt, NFIL3, and TOX, we found that only GATA3 modulated the development of ILCs. We go on to show that ectopic expression of GATA3 mRNA in CD34^+^ HSCs modulated gene expression, and significantly induced the development of CD34^+^α4β7^+^ CILPs. As a result, GATA3 significantly changed the fate of these cells by inducing the development of CD127^+^CD117^+^ early ILCPs, as well as more mature ILC populations.

The generation of CILPs from HSCs necessitate various transcription factors to act at different stages of development. The role of transcription factors including, but not limited to, Bcl11b, GATA3, ID2, NFIL3, PLZF, RORα, RORγt, Tbet, TCF7, and TOX during ILC development have been reported (19, 20, 22, 23, 28, 40, 41). It was somewhat surprising to observe that transfection of a number of these transcription factors (ID2, NFIL3, RORγt, and TOX) had no significant effect on ILC development when transfected into CD34^+^ cells. However, these data likely point to the critical nature of temporal orchestration and perhaps transcription factor dose for lineage specification (42). In contrast to our results Nagasawa and colleagues used lentivirus transduction of ID2 in thymus-derived CD34^+^ cells and showed that ID2 led to the development of CD5^+^ immature ILC progenitors by repressing T cell development (43). While it is difficult to directly reconcile their findings with our results, where ID2 had no significant influence on ILC lineage specification or development, salient differences between the two studies include the stem cell source used (cord blood vs. thymic CD34^+^ cells) and method of gene expression (transient mRNA vs. enforced lentivirus expression). Since the initiation of the studies presented here, several recent murine studies demonstrate GATA3 to be a master transcription regulator, not only for ILC2 development, but also for all helper ILCs (19, 28, 44, 45). In support of this, GATA3 deficient mice show dramatic reductions in the proportion of ILC progenitors (27, 28). Our results using human CD34^+^ cells and transient GATA3 mRNA transfection align with these results and perhaps extend them, as we show that GATA3 increases in the CD34^+^α4β7^+^ fraction, known to give rise to ILCs (12, 20, 28).

We also find that transfection of HSCs with GATA3 mRNA diminishes NK cell differentiation by enhancing the development of other helper ILCs. Despite the inability of EL08.1D2 feeder cells to support ILC2 development, our single cell cloning experiments demonstrated that GATA3 increases HSC differentiation into the ILC3 lineage, while reducing the proportion of cells committed to NK cell lineage. Thus, these findings show that GATA3 directly influences the generation of ILCs by acting at multiple stages of HSC development. The first stage is the premature stage where GATA3 acts on HSCs with multi-lineage potential to enhance the generation of early ILC committed progenitors marked by the α4β7^+^ integrin. Second, and as supported by our single cell cloning experiments, GATA3 also acts on early ILC committed progenitors to push the development of the progenitors into the helper ILC lineages. One limitation of this work is our inability to definitively distinguish human CHILP-derived ILC1s from NK cells. Thus, we are not able to definitively state what impact GATA3 has on ILC1's, but the data presented here clearly shows that it facilitates the development of ILC2's and 3's at the cost of the bulk ILC1/NK population. Perhaps these results are consistent with GATA3 acting on a committed progenitor that gives rise to the ILC2 and 3 cells.

Our findings are in line with previous murine studies that implicate GATA3 as a critical regulator of murine CILP generation and IL-7Rα^+^ ILCs, but not NK cells development (27, 45). Among ILCs, only LTi-like cells remained unchanged in a GATA3 conditional knockout mouse whereby, ILC2 and NCR^+^ ILC3s were undetectable (27, 44). While GATA3 has been associated with the development of human ILC2s (25) our data highlight that transient expression of GATA3 in a committed ILCPs boosts the development of human ILC3 *in vitro*, supporting this established concept in murine studies (27, 45). Lastly, while these studies elucidate the role of GATA3 in human ILC and NK development, we also see these studies as the first step in a translational approach to produce ILCs for adoptive transfer prior to or after HCT or in other conditions, such as inflammatory bowel disease where the restorative action of ILCs might be beneficial. In summary, transient overexpression of GATA3 by mRNA electroporation in premature HSCs induces the generation of ILCPs, whereas high levels of GATA3 in more committed progenitor results in the skewing of cells that would typically become NK cells toward helper ILC lineage. Therefore, this study supports the findings that GATA3 induces CILPs and favors the differentiation of CILPs to become helper ILCs at the expense of NK cells.

## Data Availability

The raw data supporting the conclusions of this manuscript will be made available by the authors, without undue reservation, to any qualified researcher.

## Ethics Statement

The samples for this study were de-identified umbilical cord blood (UCB) purchased from St. Louis Cord Blood Bank. Hence, ethical review and approval was not required in accordance with the local legislation and institutional requirements.

## Author Contributions

DT designed the study, performed experiments, analyzed data, and wrote the manuscript. AY, TS, SS, JL, and RW performed experiments and edited the manuscript. GT analyzed data and edited the manuscript. KJ designed the study, analyzed data, and edited the manuscript. MV designed and directed the study, analyzed data, and wrote the manuscript.

### Conflict of Interest Statement

The authors declare that the research was conducted in the absence of any commercial or financial relationships that could be construed as a potential conflict of interest.
