# Supplementary material for: Transient Expression of GATA3 in Hematopoietic Stem Cells Facilitates Helper Innate Lymphoid Cell Differentiation
Source: Front Immunol. 2019 Mar 21;10:510. doi: 10.3389/fimmu.2019.00510 (PMC6438154; doi:10.3389/fimmu.2019.00510)
Supplement: Supplementary file 1 [file Data_Sheet_1.docx]

**Transient Expression of GATA3 in Hematopoietic Stem Cells Facilitates Helper Innate Lymphoid Cell Differentiation**

Dejene M. Tufa^1,2^, Ashley M. Yingst^1,2^, Tyler Shank^1,2^, Seonhui Shim^1,2^, George Devon Trahan^1,2^, Jessica Lake^1,2^, Renee Woods^1,2^, Kenneth L. Jones^1,2^ and Michael R. Verneris^1,2^

Affiliations:

1 University of Colorado, School of Medicine, Department of Pediatric Hematology, Oncology and BMT, Aurora, CO, USA.

2 Children’s Hospital Of Colorado, Department of Children’s Cancer and Blood Disorders, Aurora, CO, USA.

Corresponding Author:

Michael R. Verneris, MD

Pediatric Bone Marrow Transplant

University of Colorado Anschutz Medical Campus

Research Complex 1, North Tower

12800 E. 19th Ave.

Mail Stop 8302

Aurora, CO 80045

Email: Michael.Verneris@ucdenver.edu

**Supplementary Figure 1. Isolation and Expansion of Cord blood-derived CD34^+^ HSCs.** Histogram shows CD34^+^ HSCs (purity =98%) that were isolated from umbilical cord blood and expanded for 5 days (purity =99%).

**Supplementary Figure 2. Pre-sort and post-sort of CD34^+^α4β7^-^ HSCs**. Gating strategy, 5-days expanded CD34^+^ HSCs were sorted by FACS into CD34^+^α4β7^-^ and CD34^+^α4β7^+^ compartment.

**Supplementary Figure 3. Overexpression of GATA3, ID2, RORγt, NFIL3 and TOX in HSCs.** Five-day expanded UCB CD34^+^ HSCs were transfected with either GATA3, ID2, RORC, NFIL3, TOX or scrambled control mRNAs. Cells were then stained with their respective antibodies after 48 h of electroporation followed by flow cytometry analysis.

**Supplementary Figure 4.** Heat map showing log transformed differential expression for each donor pair (GATA3 versus control) for genes upregulated (A) and downregulated (B) by GATA3 in all three donors in common.

**Supplementary Figure 5. Expression of CD117 by ILCPs and ILCs.** Differentiating HSCs were stained for the surface markers CD11a, CD56, CD117 and CD336 at day 14 and 21, and dot plots are shown (n=4). Values represent the percentage of CD56^+^CD336^+^ ILCs.


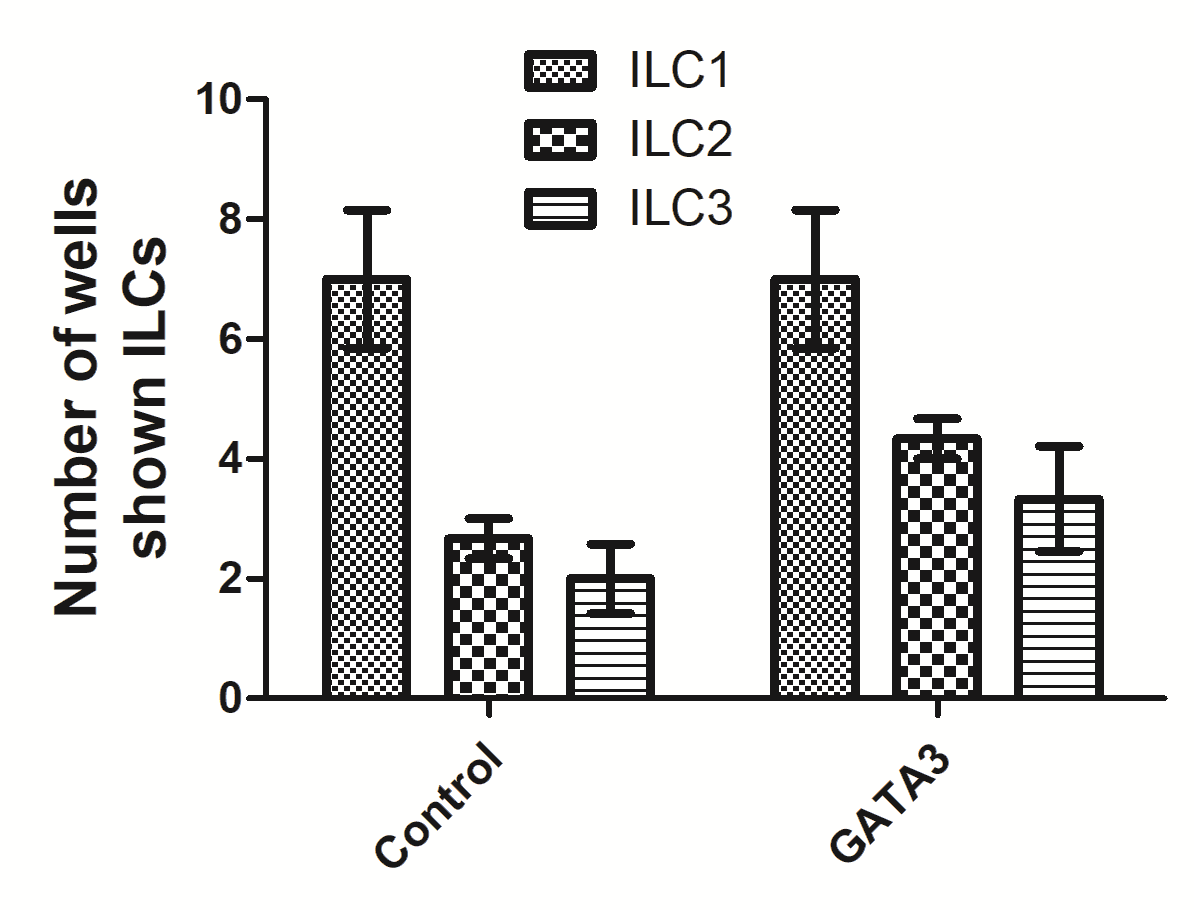


**Supplementary Figure 6. Single cell cloning demonstrated GATA3 increases ILC2 growth.** Either mock or GATA3 mRNA transfected CD34^+^ HSCs from 3 donors were single cell FACS sorted and differentiated for 28 days on pre-plated and irradiated OP9 stromal cell line in 96 well plates. Cells were stained for ILCs surface markers at day 28 of culture and the number of wells showing either CD11a^+^CD94^+^ ILC1, CD294^+^CD336^-^ ILC2 and CD11a^-^CD117^+^ ILC3 cells are shown in bar graphs (n=3).
